# Ontology-based literature mining and class effect analysis of adverse drug reactions associated with neuropathy-inducing drugs

**DOI:** 10.1186/s13326-018-0185-x

**Published:** 2018-06-07

**Authors:** Junguk Hur, Arzucan Özgür, Yongqun He

**Affiliations:** 1 Department of Department of Biomedical Sciences, University of North Dakota School of Medicine and Health Sciences, Grand Forks, ND 58202 USA; 2 Department of Computer Engineering, Bogazici University, 34342 Istanbul, Turkey; 3 Unit for Laboratory Animal Medicine, Department of Microbiology and Immunology, University of Michigan Medical School, Ann Arbor, MI 48109 USA; 4 Department of Microbiology and Immunology, University of Michigan Medical School, Ann Arbor, MI 48109 USA; 5 Center for Computational Medicine and Bioinformatics, University of Michigan Medical School, Ann Arbor, MI 48109 USA; 6 Comprehensive Cancer Center, University of Michigan Medical School, Ann Arbor, MI 48109 USA

## Abstract

**Background:**

Adverse drug reactions (ADRs), also called as drug adverse events (AEs), are reported in the FDA drug labels; however, it is a big challenge to properly retrieve and analyze the ADRs and their potential relationships from textual data. Previously, we identified and ontologically modeled over 240 drugs that can induce peripheral neuropathy through mining public drug-related databases and drug labels. However, the ADR mechanisms of these drugs are still unclear. In this study, we aimed to develop an ontology-based literature mining system to identify ADRs from drug labels and to elucidate potential mechanisms of the neuropathy-inducing drugs (NIDs).

**Results:**

We developed and applied an ontology-based SciMiner literature mining strategy to mine ADRs from the drug labels provided in the Text Analysis Conference (TAC) 2017, which included drug labels for 53 neuropathy-inducing drugs (NIDs). We identified an average of 243 ADRs per NID and constructed an ADR-ADR network, which consists of 29 ADR nodes and 149 edges, including only those ADR-ADR pairs found in at least 50% of NIDs. Comparison to the ADR-ADR network of non-NIDs revealed that the ADRs such as pruritus, pyrexia, thrombocytopenia, nervousness, asthenia, acute lymphocytic leukaemia were highly enriched in the NID network. Our ChEBI-based ontology analysis identified three benzimidazole NIDs (i.e., lansoprazole, omeprazole, and pantoprazole), which were associated with 43 ADRs. Based on ontology-based drug class effect definition, the benzimidazole drug group has a drug class effect on all of these 43 ADRs. Many of these 43 ADRs also exist in the enriched NID ADR network. Our Ontology of Adverse Events (OAE) classification further found that these 43 benzimidazole-related ADRs were distributed in many systems, primarily in behavioral and neurological, digestive, skin, and immune systems.

**Conclusions:**

Our study demonstrates that ontology-based literature mining and network analysis can efficiently identify and study specific group of drugs and their associated ADRs. Furthermore, our analysis of drug class effects identified 3 benzimidazole drugs sharing 43 ADRs, leading to new hypothesis generation and possible mechanism understanding of drug-induced peripheral neuropathy.

## Background

While drugs have been widely and successfully used to treat various diseases, most drugs cause different adverse events (AEs), commonly called adverse drug reactions (ADRs). These ADRs are sometimes severe and significantly affect public health. Indeed, ADRs are listed as the fourth killer after heart disease, cancer, and stroke [1]. Therefore, it is critical to carefully study the ADRs and underlying mechanisms.

Multiple studies have been conducted to automatically identify ADRs in text using Natural Language Processing (NLP) techniques. Different types of data sources such as electronic health records [2], scientific publications, and social media data have been used to extract ADRs. A lexicon of ADR-related terms and concepts was compiled from different sources such as the Unified Medical Language System (UMLS) [3] and the side effect resource (SIDER) [4] and was used to match the ADR mentions in user comments retrieved from DailyStrength (http://www.dailystrength.org) by Leaman et al. [5]. Nikfarjam and Gonzalez used the same user comment data set and developed an association rule mining approach to tag ADR mentions [6]. Similarly to Leaman et al., Gurulingappa et al. [7] also developed a lexicon-based matching approach to identify ADRs in text using the lexicon created based on the Medical Dictionary for Regulatory Activities (MedDRA) [8] and DrugBank [9]. However, rather than using user comments from social media, Gurulingappa et al. used the abstracts of case reports as their data source. Product labels have also been used as data sources to extract ADRs and create knowledge bases of known ADRs [10, 11]. A review of recent techniques on ADR extraction from text from various data sources is available in [12].

An important group of ADRs is neuropathy. Using FDA reported package insert documents and drug safety records, our previous studies identified 242 neuropathy-inducing drugs (NIDs) through mining various public resources and drug labels [13, 14]. We have previously developed an Ontology of Drug Neuropathy Adverse Events (ODNAE) that ontologically represents 214 NIDs, corresponding chemicals of these drugs, chemical function, adverse events associated with these drugs, and various other chemical characteristics [14]. Our study also showed that ODNAE provides an ideal platform to systematically represent and analyze AEs associated with neuropathy-inducing drugs and generate new scientific insights and hypotheses [14]. One weakness of the ODNAE study is that ODNAE only collects neuropathy-related ADRs commonly found in drug package insert documents and misses the collection of non-neuropathy ADRs from different sources.

In addition to enhanced literature mining, ontology can also be used for advanced class effect analysis. Specifically, an AE-specific drug class effect is defined to exist when all the drugs in a specific drug class (or drug group) are associated with an AE. In a recent study on cardiovascular drug-associated AEs, a proportional class-level ratio (PCR) value was defined and used to identify drug class effect on different AEs [15]. Specifically, when the PCR value equals to 1, it means that a class effect of a group of drugs on a specific AE exists. Previous PCR-based heatmap analyses identified many important drug class effects on different AEs [15].

In addition to the official FDA drug package insert documents, FDA also collects large amounts of spontaneous ADR case reports. To better understand these case report data, it is critical to use standardized terminologies or ontologies to identify drugs, ADRs, and associated data from the text reports. Therefore, ontology-based literature mining becomes critical. Previously, we applied the Vaccine Ontology (VO) [16] to enhance our literature mining of interferon-gamma related [17], *Brucella*-related [18], and fever-related [19] gene interaction networks in the context of vaccines and vaccinations. In these studies, we used and expanded SciMiner [20], a literature mining program with a focus on scientific article mining. SciMiner uses both dictionary- and rule-based strategies for literature mining [20].

To better study biological interaction networks, we have also developed a literature mining strategy CONDL, or Centrality and Ontology-based Network Discovery using Literature data [19]. The centrality analysis here refers to the application of different centrality measures to calculate the most important genes (i.e., hub genes) of the resulting gene-gene interaction network out of biomedical literature mining. Centrality measures, including degree, eigenvector, closeness, and betweenness, have been studied [19, 21]. The CONDL strategy was applied to extract and analyze IFN-γ and vaccine-related gene interaction network [21] and vaccine and fever-related gene interaction network [19], and our results showed that centrality analyses could identify important genes and raise novel hypotheses based on literature mined gene interaction networks.

The main purpose of this study was to develop a CONDL method for literature mining of all ADRs associated with neuropathy inducing drugs (NIDs) and used the mined results for systematic network and class effect analyses. Using MedDRA [8], ODNAE [14], Chemical Entities of Biological Interest (ChEBI) [22], and Ontology of Adverse Events (OAE) [23], we developed an ontology-based ADR-SciMiner tool for identifying ADRs from drug labels and applied it to NIDs to ontologically model their ADR-associated characteristics. The literature mined results were then used for ontology-based class effect analysis, leading to new scientific discoveries.

## Methods

**Fig. 1:**
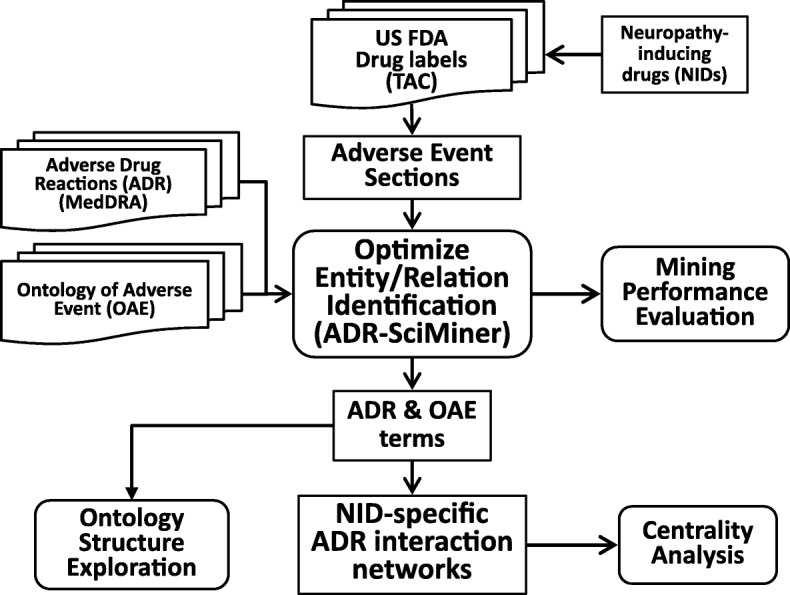
Project workflow. This figure illustrates our overall workflow in the present study. US FDA drug labels were analyzed to identify ADRs and normalized them through MedDRA v20 and OAE using ADR-SciMiner. A network of ADR-ADR based on the ADRs reported to have been caused by NIDs was constructed. The most central ADRs in the network were analyzed. The characteristics of NID-associated ADRs were further explored using the ontological structures in OAE

The overall workflow of our ontology-based literature mining approach for the study of neuropathy-inducing drugs (NIDs) is illustrated in Fig. 1. Briefly, our approach included development of ADR-SciMiner platform that identifies ADRs from drug labels using the terms in MedDRA and OAE. Various term expansion, name matching, and filtering rules have been implemented. The mining performance was evaluated using manually curated drug labels. The final version of ADR-SciMiner was applied to the NID labels and the results were examined using the ADR-ADR interaction network and the OAE hierarchical structure.

### NID drug labels

**Fig. 2:**
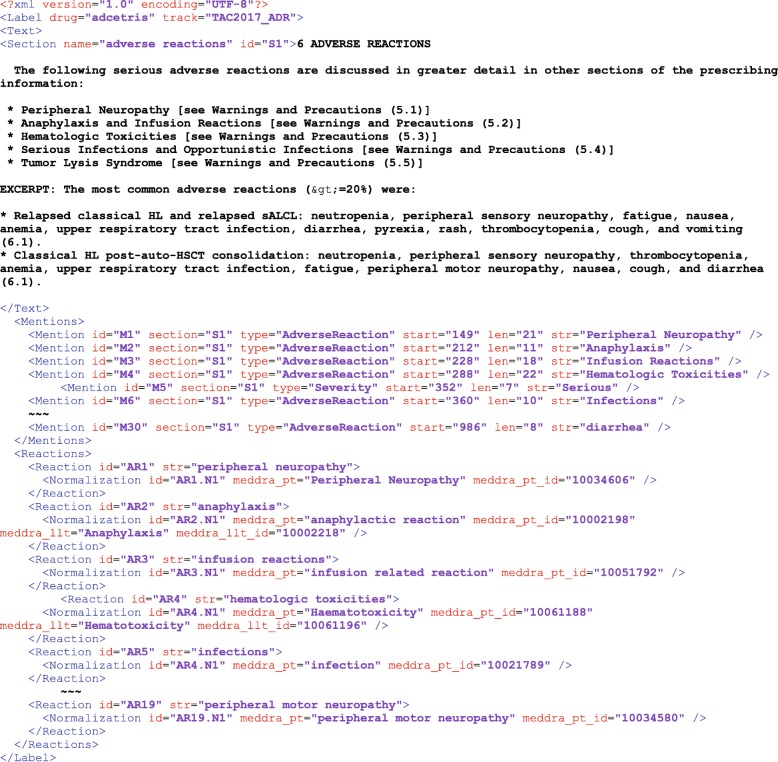
XML-formatted drug label. This figure illustrates an example of XML-formatted drug labels (adcetris) from the training set. The content has been reduced and simplified to fit into a figure for demonstration purpose. Typical XML-formatted labels from the training set include three main sections: “Text” containing the texts from ADR-relevant sections from drug labels; “Mentions” containing the manually curated ADRs; and “Reactions” containing normalized ADRs in terms of MedDRA terms

In the present study, we used a collection of XML-structured drug labels that are applied for the Text Analysis Conference (TAC) Adverse Drug Reaction Extraction from Drug Labels track (https://tac.nist.gov/2017/). This data set includes the adverse event sections from a total of 2308 US FDA drug labels, which were split into two sets: *Training* set and *Unannotated* set, each containing 101 and 2207 drug labels. The *Training* set contained manually curated ADRs provided by the TAC organizing committee. Among 2207 drug labels in the *Unannotated* set, TAC provided 99 labels with manually curated ADRs, which were used for performance evaluation of ADR-SciMiner. Figure 2 illustrates an example of XML-formatted drug-label from the *Training* set.

NIDs were collected from our previous two studies: one examining the systems pharmacological aspects of NIDs [13] and another focusing on ontology-based collection, representation and analysis of drug-associated neuropathy adverse events [14].

### SciMiner tagging of ADR and drug terms

SciMiner was originally developed as a web-based literature mining platform, designed for identification of human genes and proteins in a context-specific corpus [20]. Later, SciMiner was updated to identify bacterial genes and various biomedical ontologies such as Vaccine Ontology (VO) and Interaction Network Ontology (INO), developed by our groups, resulting in specific variations of SciMiner: INO-SciMiner [24], VO-SciMiner [18], and E-coli-SciMiner [25]. In this study, we developed another version of SciMiner, specializing in the identification and analysis of ADRs from the US FDA drug labels.

MedDRA, or Medical Dictionary for Regulatory Activities, is a clinically validated standardized medical terminology dictionary (and thesaurus), consisting of five levels of hierarchy. MedDRA has been widely used for supporting ADR reporting in clinical trials [8, 26]. MedDRA release version 20 (https://www.meddra.org/) and the OAE ontology were used as the source of the ADR terms, which have been incorporated into SciMiner dictionary for ADR term identification. Perl package Lingua::EN was used to expand the ADR dictionary allowing the inclusion of additional plural or singular forms where only one form is included in the dictionary. For example, ‘peripheral neuropathy’ has been expanded to include ‘peripheral neuropathies’. Besides, various term variation and filtering rules were implemented to improve the accuracy of ADR term tagging. For example, MedDRA terms ID 10003481 has preferred name of ‘Aspartate aminotransferase increased’. ADR-SciMiner was designed to properly identify variations of this preferred name such as ‘increased AST’, ‘AST elevated’, and ‘high AST’. To reduce false positives, any matching ADR terms from section or table headers of drug labels were excluded.

### Performance evaluation of ADR-SciMiner

The TAC dataset included 200 manually curated labels (101 in the *Training* and 99 in the *Unannotated* sets) and the details have been recently published [27]. Briefly, four annotators, including two medical doctors, one medical librarian and one biomedical informatics researcher, participated in the manual annotation process of these 200 drug labels. These annotators were all trained biomedical annotation and the drug labels were annotated independently by these annotators. Any disagreements were reconciled in pairs or collectively resolved by all four annotators. The mining performance of ADR-SciMiner was evaluated using the 99 drug labels in the *Unannotated* set. The evaluation was done at the level of normalized MedDRA Preferred Terms (PTs) for each drug. Recall, Precision, and F-Score were calculated.

### Generation of ADR-ADR network and its analysis

NID and non-NID associated ADR-ADR networks were constructed in our study. ADRs were represented as the nodes of the network. Two nodes were connected by an edge if they are associated with the same drug. In order to obtain highly prevalent NID and non-NID specific ADRs, an edge weight threshold of 50% was set. In other words, two ADRs were connected by an edge if they co-occur together as ADRs of at least 50% of the NID or non-NID drugs. Centrality analysis was performed on the ADR-ADR networks using the Cytoscape plug-in CentiScaPe [28] to identify the most salient NID and non-NID associated ADRs. Degree centrality and eigenvector centrality were computed. Degree centrality corresponds to the number of neighbors a node has. Each neighbor contributes equally to the centrality of the node. On the other hand, in eigenvector centrality the contribution of each neighbor is proportional to its own centrality.

### ChEBI and OAE-based ontological analyses of three neuropathy-inducing drugs and associated ADRs

The drugs were mapped to ChEBI [22] terms, which are also imported and used in the ODNAE. The identified ADRs were mapped to OAE terms, and the OAE structure was used to classify and analyze the ADR structure. To extract the associated drugs, AEs, and their related terms, the Ontofox tool [29] was used. The Protégé OWL editor [30] was used to visualize the hierarchical structure of these extracted terms.

### Ontology-based analysis of drug class effects on AEs

ChEBI was used to classify NIDs into different higher-level classes or groups. For each high or intermediate level class, we calculated the drug class effect on AEs. Specifically, all the identified 53 NIDs were classified into different categories using ChEBI. The AEs associated with each NID were identified in the previous studies. Based on these results, we were able to identify the common AEs associated with all NIDs under a specific class (e.g., benzimidazole drugs). Based on the class effect definition, these results indicate that there exists a class effect of the specific class on the common AEs (i.e., the PCR value =1) [15]. All the common AEs were then classified based on OAE using the Ontofox tool [29].

## Results

### NID drug labels

From our two published studies on neuropathy-inducing drugs [13, 14], we collected a total of 242 NIDs. We also obtained a collection of XML-structured drug labels that are used for the 2017 Text Analysis Conference (TAC) Adverse Drug Reaction Extraction from Drug Labels track. This data set contains the adverse event sections of a total of 2308 US FDA drug labels in two subsets: *Training* set with 101 labels and *Unannotated* set with 2207 labels, which corresponded to a total of 1883 unique drugs. There were 299 unique drug names, each of which included two or more labels, because a drug in our study refers to a generic drug name or an active drug ingredient which can have multiple brands with different labels. Among the 2308 labels, there were 69 labels corresponding to 53 NIDs, which served as the dataset in the present study.

### SciMiner tagging of ADR and drug terms and performance evaluation

ADR-SciMiner has been developed to include the dictionary of ADRs based on MedDRA release 20 and the current version of OAE. The ADR term dictionary is expanded to include variations such as plural vs singular nouns to increase the coverage. The performance of current version of ADR-SciMiner was evaluated based on the ADRs from 99 labels. These labels included 5158 MedDRA PT terms, while ADR-SciMiner reported 5360 PT terms collectively. ADR-SciMiner correctly identified 4198 of these 5158 PTs in the TAC data: a recall of 0.81, a precision of 0.75, and an F-Score of 0.77 was obtained.

### MedDRA representation of ADRs

Table 1 summarizes the numbers of identified ADRs from the 53 NIDs. These NIDs are a subset of the total NIDs identified in our previous studies [13, 14]. We did not use all the over 200 NIDs because only these 53 NIDs have corresponding ADR text data in the FDA TAC 2017 dataset. Briefly, ADR-SciMiner identified approximately an average of 243 ADRs per drug (114 unique ADRs per drug). Antidepressant medicine Venlafaxine had the most ADRs of 433, while glucocorticoid triamcinolone has the least ADRs of 9 (Table 1).

**Table 1:**
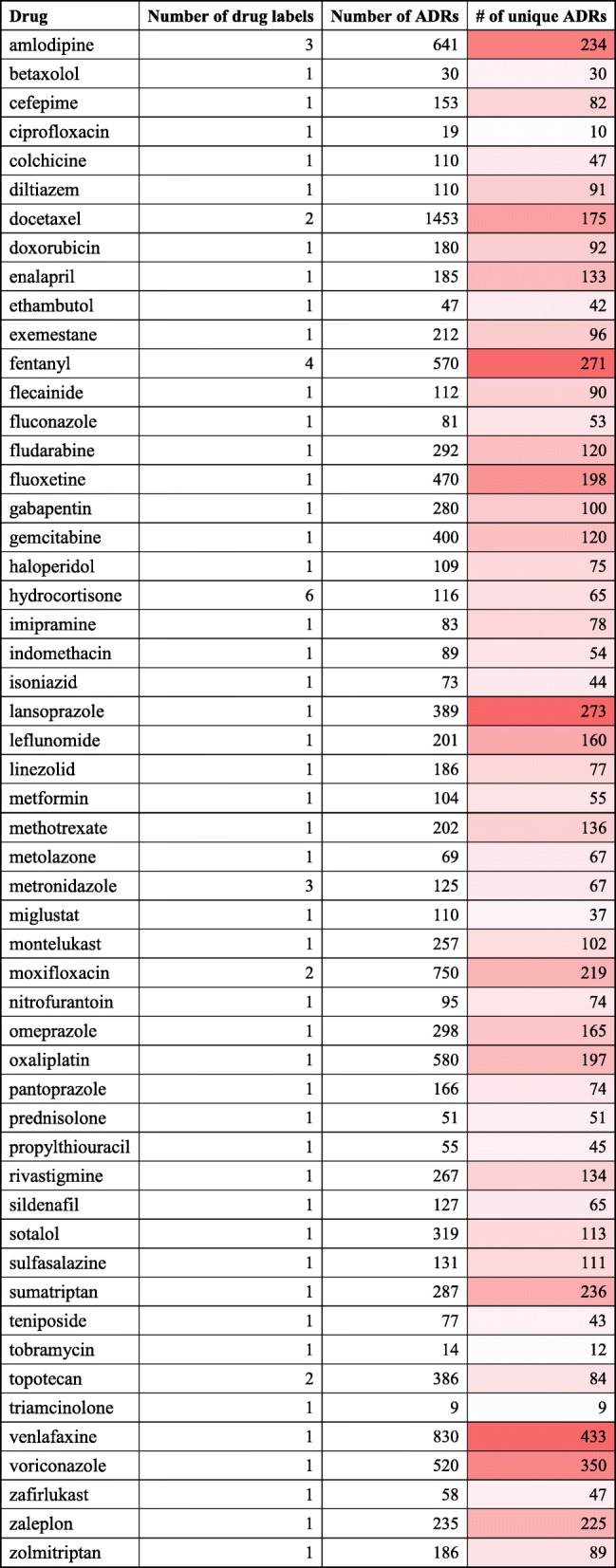
Identified ADRs from 53 NIDs drug labels

Color highlight was used to visualize difference among the number of ADRs across NIDs

### Literature mining statistics and ADR-ADR network

**Fig. 3:**
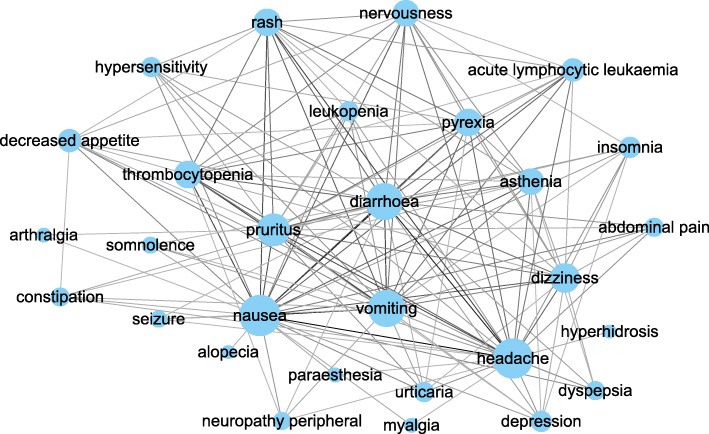
NID associated ADR network. Two ADRs are connected by an edge if they co-occur in over 50% of the NIDs. Node sizes are proportional to the degrees of the nodes. Edge thickness corresponds to the number of drugs having two ADRs

Figure 3 is a NID-associated ADR network based on the cutoff of co-occurrence of two ADRs connected in at least 50% (i.e., 27 out of 53) of the NIDs. The NID specific ADR-ADR network shown in Fig. 3 contains 29 nodes and 149 edges. The common ADRs are located at the center of the network, including terms like headache, vomiting, pyrexia, nausea, dizziness, etc. More specific analysis of the network is reported below.

### Centrality analysis of ADR-ADR network

The eigenvector and degree centrality scores of the 29 ADRs found using NIDs are shown in Table 2. The same approach was used to construct a non-NID specific ADR-ADR network, where two ADRs are connected by an edge if they co-occur in at least 50% of the remaining (i.e., non-NID drugs). This resulted in a network containing only six ADRs, namely headache, vomiting, diarrhoea, rash, nausea, and dizziness. Although these are also among the most central ADRs in the NID specific network, they are not NID specific, since they are also prevalent and commonly occur together in the non-NID case. Some notable ADRs central in the NID-specific network but not parts of the non-NID specific network include pruritus, pyrexia, thrombocytopenia, nervousness, asthenia, acute lymphocytic leukaemia, decreased appetite, insomnia, and depression. Degree and eigenvector centrality produced the same ranking (Table 2).

**Table 2:** The centrality scores of the ADRs in the NID specific ADR-ADR network

ADR	Degree	Eigenvector
nausea	27	0.311
headache	26	0.310
vomiting	23	0.301
diarrhoea	23	0.301
pruritus	19	0.270
dizziness	16	0.245
pyrexia	14	0.231
rash	14	0.231
thrombocytopenia	14	0.228
nervousness	13	0.222
asthenia	13	0.214
acute lymphocytic leukaemia	10	0.187
decreased appetite	10	0.177
insomnia	8	0.149
depression	8	0.148
urticaria	7	0.139
hypersensitivity	7	0.138
leukopenia	7	0.137
abdominal pain	6	0.122
dyspepsia	6	0.118
constipation	6	0.114
neuropathy peripheral	5	0.102
seizure	4	0.086
somnolence	4	0.086
paraesthesia	2	0.044
myalgia	2	0.044
arthralgia	2	0.041
alopecia	1	0.022
hyperhidrosis	1	0.022

Two centrality measures (degree and eigenvector) were calculated using Cytoscape app CentiScaPe

### Ontology-based analysis of benzimidazole NID drugs and their associated ADR types

**Fig. 4:**
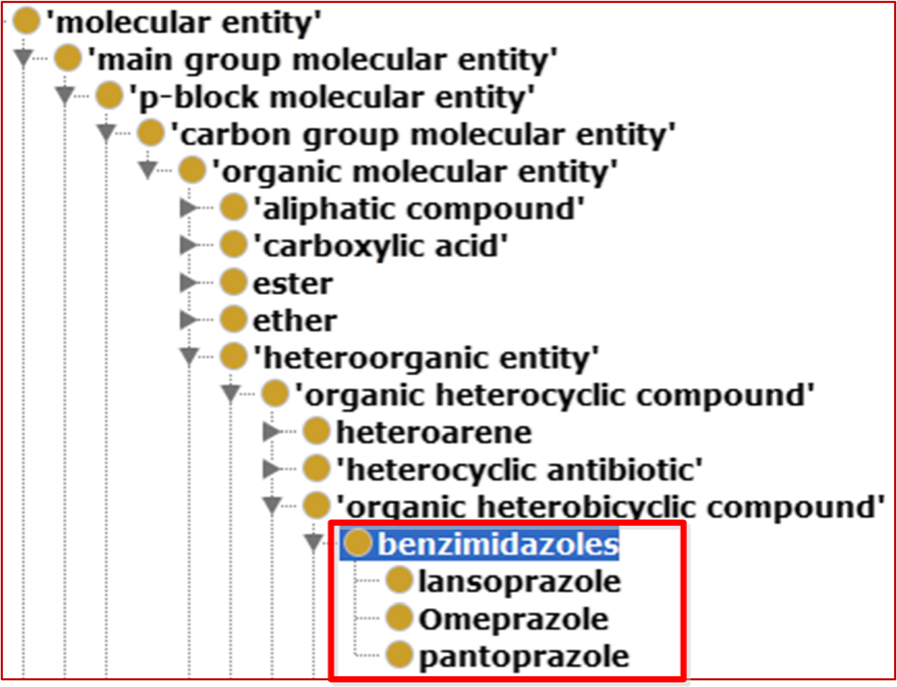
Identification of three benzimidazole drugs associated with neuropathy adverse events. The three drugs were grouped by ChEBI under the benzimidazoles chemical group. The hierarchical structure of the benzimidazoles chemical group is also laid out

Out of the 53 drugs, we used the ChEBI chemical ontology structure to examine the chemical classification of these 53 drugs and their associated upper-level hierarchies. One interesting group of chemicals becomes interesting to us, which is the group of benzimidazole, a colorless heterocyclic aromatic organic compound that consists of the fusion of benzene and imidazole [31]. Benzimidazole drugs are structural isosteres of naturally-occurring nucleotides, allowing them to interact with the biopolymers of living systems and become an important group of drugs with antimicrobial, anti-inflammatory, and anticancer activities. The three benzimidazole NIDs identified in our study include lansoprazole, omeprazole, and pantoprazole (Fig. 4), which are all proton-pump inhibitors that inhibit gastric acid secretion [32]. These three drugs can all be used for relief of symptoms of gastroesophageal reflux disease, gastric and duodenal ulcer disease, and eradication of *Helicobacter pylori* infection [32]. Their shared and different ADR profiles have not been studied.

**Fig. 5:**
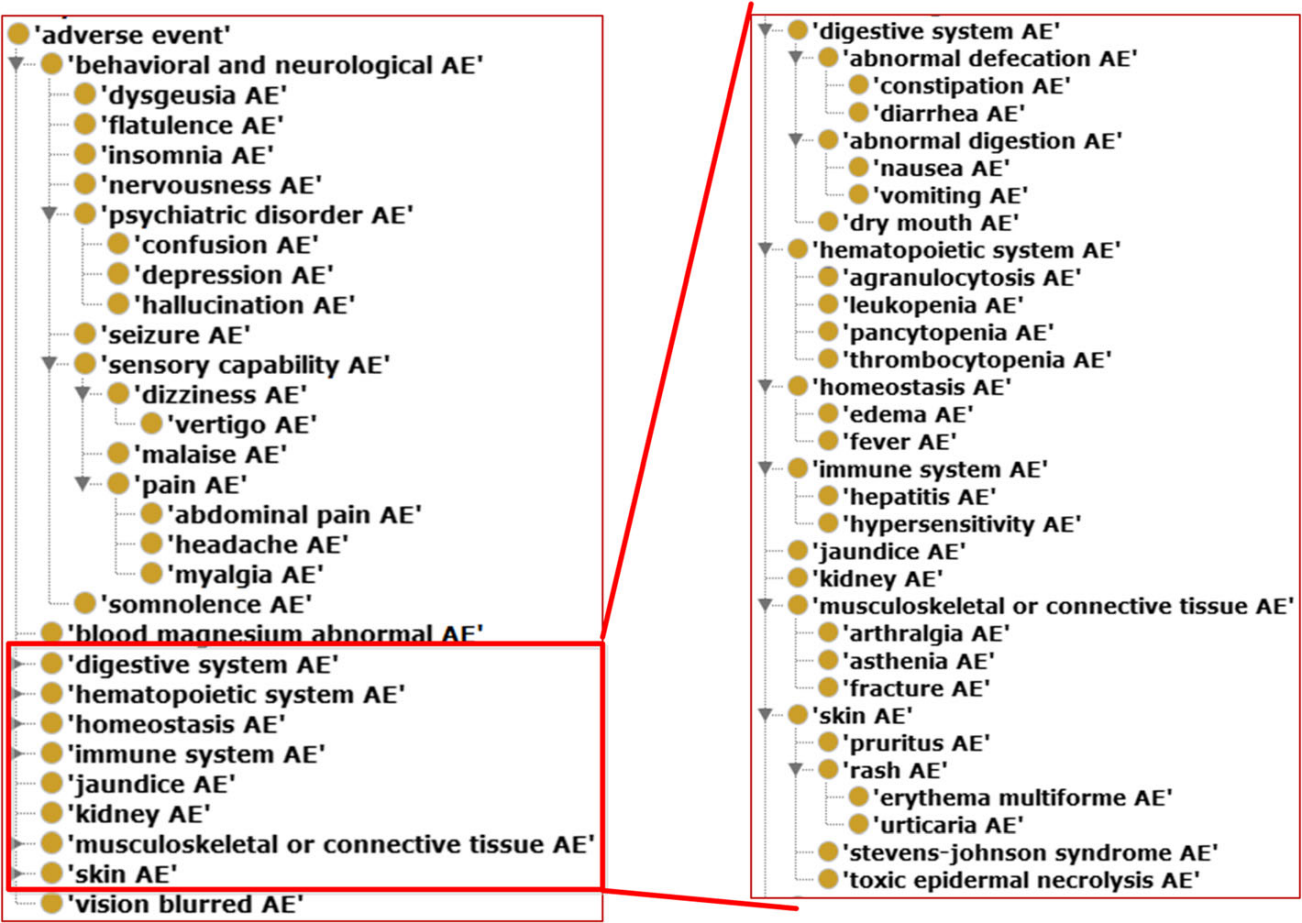
Hierarchical display of 43 ADRs associated with three benzimidazoles drugs. The OAE IDs corresponding to the 43 ADRs were identified, and Ontofox was used to these terms and their associated hierarchical terms using the “IncludeComputedIntermediate” condition

In our study, lansoprazole, omeprazole, and pantoprazole are associated with 389 (273 are unique), 298 (165 are unique), and 166 (74) ADRs, respectively. We identified 43 ADRs associated with all three drugs. Based on our drug class effect definition [15], these 43 ADRs are all categorized as AEs out of the class effect of the benzimidazole drug class. Furthermore, we applied the OAE to generate a subset view of these ADRs in the OAE framework (Fig. 5). As shown in this figure, these 43 ADRs are focused on behavioral and neurological ADRs, digestive ADRs, and skin ADRs. There are also many ADRs in the hematopoietic system, homeostasis system, immune system, and muscular system.

## Discussion

The contributions of this study are multiple fold. First, we developed and applied an ontology-based SciMiner literature mining approach, which was then used to mine the FDA TAC 2017 dataset. It is a huge challenge to identify all ADRs using textual description of ADR case reports. Our MedDRA/OAE-based SciMiner literature mining approach was successfully used to mine the FDA TAC 2017 dataset with a special focus on 53 neuropathy-inducing drugs (NIDs). Our study demonstrates the important role of the MedDRA controlled terminology and ontologies (e.g., ChEBI, OAE, and ODNAE) in the literature mining and further ADR analysis. Second, we constructed an ADR-ADR network and applied centrality analysis to identify the hub ADRs in the network. Third, among the 53 NIDs, our ChEBI-based analysis found three benzimidazole drugs, which formed a drug class effect on 43 ADRs. An OAE analysis of these ADRs further identified many enriched ADR categories. Based on the results, we can hypothesize that the proton-pump inhibition role, common to all the three benzimidazole drugs, might participate in different pathways leading to these ADRs. To our knowledge, our study represents the first of such literature mining-derived ontology-based drug class effect analysis.

The present study is based on a subset of US FDA drug labels, which was included in the 2017 Text Analysis Conference (TAC) Adverse Drug Reaction Extraction from Drug Labels track. We used this data set as a proof of concept as well as to develop a prototype version of ADR-SciMiner. We assumed that if an ADR is mentioned in the file of a drug, it is associated with the drug. However, it is likely that the ADR occurs within a negation or speculation statement such as ‘depression was not observed as an ADR of the drug’ or ‘depression might be an ADR of the drug’. Therefore, more semantic oriented NLP analysis techniques may be developed to identify whether an ADR is really associated with a drug or not.

To identify the most salient ADRs associated with NIDs, we created ADR-ADR networks both specific to NIDs and non-NIDs using a threshold of 50% for association. In other words, two ADRs were connected by an edge, if they co-occur in at least 50% of the NIDs or non-NIDs. Six of the central ADRs in the NID specific network were also included in the non-NID specific network, showing that these are prevalent and commonly occur together both in NID and non-NID cases. The other ADRs in Table 2 are central only in the NID associated network, which might reveal that they are more NID specific. As future work, we plan to extend the network analysis by including the specific drugs to the network as well and creating bipartite drug-ADR networks. The types of relations between drugs and ADRs can be identified by using the Interaction Network Ontology (INO) [24].

Our study identified three benzimidazole drugs (i.e. lansoprazole, pantoprazole, and omeprazole) that induce similar profiles of ADRs. Overall these three drugs have been found safe in terms of their associated ADR reports [33–35]. For example, a previous study with 10,008 users of lansoprazole in daily practice indicated that the most frequently reported lansoprazole ADRs were diarrhoea, headache, nausea, skin disorders, dizziness, and generalized abdominal pain/cramps, but no evidence of rare ADRs were found [33]. Current study found many ADRs associated with each of these three drugs, and all these three drugs are associated with 43 ADRs, commonly behavioral and neurological, digestive, muscular, and skin ADRs. A common reason for stopping pantoprazole usage was found to be the diarrhea ADR [34], which is also listed as one of the 43 ADRs.

A previous study suggested that these three drugs have similar profiles to interact with other drugs (most commonly vitamin K antagonist), suggesting a class effect [36]. According to the ODNAE records [14], lansoprazole, omeprazole, and pantoprazole are all associated with neuropathy adverse events. Our study found 43 AEs commonly shared with these three benzimidazole drugs. Interestingly, many of these AEs are also found to be the hubs of the highly enriched NID network from our literature mining data centrality analysis. It is likely that these three benzimidazole drugs, which function as proton-pump inhibitors, use the same or similar pathways to induce neuropathy adverse events.

It is noted that the ontology-based drug class effect study is novel in many aspects compared to its original report [15]. First, compared to the previous report using the drug package insert information, our study uses the data generated from literature mining of FDA provided case report data. Second, given the large size of AE data for each vaccine, we were able to identify many AEs commonly used by a class of drugs, in our case, 43 AEs associated with the three benzimidazole drugs. Our OAE-based analysis was able to further identify the common patterns among these AEs. Such a high throughput study was not reported in the previous package insert document-based studies.

The ADR identification performance is not yet optimal and there is still much room for improvement. The majority of falsely identified ADR terms by SciMiner could be grouped into three types: (1) incorrect mapping of acronyms to ADRs (e.g., ‘all’, as in ‘all patients’, mapped to ‘acute lymphocytic leukaemia’); (2) ADR that may not be caused by the current drug (e.g., ‘caution is needed in patients with diabetes’); and (3) ADRs that occur as discontinuous entities in text (e.g., ‘corneal ulceration’ is an ADR, but does not occur as a continuous text fragment in ‘corneal exposure and ulceration’). Integration of other dictionaries such as SNOMED CT [37] into ADR-SciMiner will be explored to possibly expand the ADR dictionary thus to improve the recall. Identifying whether a term is an acronym for an ADR or not, determining whether an ADR that occurs in a drug label is really caused by that drug, and detecting ADRs that occur as discontinuous text fragments in text require deeper semantic understanding of the sentences by considering the context information (i.e., the surrounding words) of an ADR in text. Our current method is a dictionary and rule-based method, which does not consider the context of an ADR occurrence in text. These challenges can be tackled by using machine learning methods with features that capture context information and utilize the syntactic analysis of the sentences such as their dependency parses.

As future work, we plan to develop machine learning based methods to improve the accuracy of ADR tagging as well as the detection of the associations between ADRs and drugs. We will also extend our approach to include all available structured drug labels in the DailyMed database, maintained by National Institute of Health. DailyMed currently contains listings of 95,513 drugs submitted to the US FDA, about 28,000 of which are prescription drugs for human. Our ontological study of NIDs will be extended using this larger drug label dataset.

## Conclusions

In this study we developed an MedDRA and ontology-based SciMiner literature mining pipeline, applied the pipeline to mine a FDA text set for ADRs associated with neuropathy-inducing drugs, performed centrality network analysis, and drug class effect studies. Our approach identified scientific insights regarding these drug-specific ADRs. Our study demonstrates the feasibility of using ontology-based literature mining, network analysis, and drug class effect classification to efficiently identify and study specific drugs and their associated ADRs.

## Abbreviations

ADR: Adverse Drug Reaction
ChEBI: Chemical Entities of Biological Interest
CONDL: Centrality and Ontology-based Network Discovery using Literature data
INO: Interaction Network Ontology
MedDRA: Medical Dictionary for Regulatory Activities
NID: Neuropathy Inducing Drug
NLP: Natural Language Processing
OAE: Ontology of Adverse Events
ODNAE: Ontology of Drug Neuropathy Adverse Events
PCR: Proportional Class level Ratio
TAC: Text Analysis Conference
VO: Vaccine Ontology
